# Neutrophils-astrocyte interactions in central nervous system inflammation

**DOI:** 10.1038/s41419-025-07945-x

**Published:** 2025-08-25

**Authors:** Bingyou Yuan, Xian Zhang, Liang Liu, Yan Chai, Jianning Zhang, Xin Chen

**Affiliations:** 1 Department of Neurosurgery, Tianjin Medical University General Hospital, Tianjin, PR China; 2 Tianjin Neurological Institute, Key Laboratory of Post-Trauma Neuro-Repair and Regeneration in Central Nervous System, Ministry of Education, Tianjin, PR China

**Keywords:** Neuroimmunology, Cell death in the nervous system

## Abstract

The concept of central nervous system (CNS) “immune privilege” has undergone substantial revision. We now understand that the CNS exhibits sophisticated inflammatory responses that serve dual functions: potentially detrimental in acute phases while facilitating repair and recovery during chronic stages of various neurological conditions. Recent advances in genomic technologies, particularly high-throughput single-cell RNA sequencing (scRNA-seq) and spatial transcriptomics, have revolutionized our understanding of cellular dynamics and interactions within the CNS inflammatory microenvironment. Here, we examine the intricate interplay between neutrophils and astrocytes during CNS inflammation. We synthesize emerging evidence of their reciprocal regulation, analyze their roles in neurological diseases, and delineate the molecular pathways mediating their communication. Understanding these cellular interactions could reveal promising therapeutic targets for modulating secondary CNS inflammation, potentially leading to more effective treatment strategies for neurological disorders.

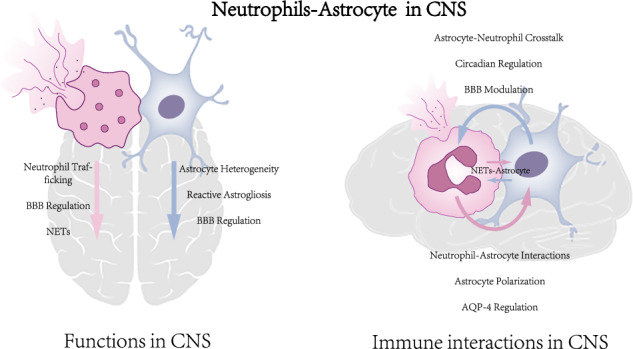

## Facts

- Neutrophils are important responders in inflammation of the CNS and are involved in regulating the immune response of the CNS.
- Astrocytes are resident cells of the CNS and are involved in regulating the inflammatory response of the CNS.
- A better understanding of neutrophil-astrocyte interrelationships could help contribute to a more comprehensive therapeutic strategy for secondary CNS inflammation.

## Open questions

- What is the role of neutrophils and astrocytes in the CNS?
- How reactive astrocytes chemotactically recruit and regulate neutrophils?
- How neutrophils regulate reactive astrocytes?
- What is the relationship between NETs and reactive astrocytes?

## Introduction

The CNS relies on intricate cellular interactions for its function, with emerging evidence highlighting crucial bidirectional communication between CNS-resident cells and the immune system. This communication is fundamental for CNS homeostasis, immune surveillance, and response to injury. At this neuroimmune interface, astrocytes—the most abundant glial cells in the CNS—and neutrophils—the predominant peripheral immune cells—emerge as key players, yet their interactions remain poorly understood. Recent advances in understanding these astrocyte-neutrophil interactions have revealed their potential significance in both CNS health and disease, prompting renewed interest in this critical cellular dialogue.

Neutrophilic granulocytes (neutrophils) represent the most abundant immune cell population in the human body, serving as critical mediators of immune defense and inflammation regulation. In humans, these cells constitute 50–70% of circulating leukocytes, while in mice, they comprise 10–25% of the circulating leukocyte population [1]. As essential components of the innate immune system, neutrophils act as first-line responders to tissue injury in both peripheral tissues and the CNS [2]. The significance of neutrophil-CNS interactions has become increasingly apparent [3], with mounting evidence highlighting their crucial role in neuroinflammation across various CNS pathologies, including Stroke, Traumatic brain injury(TBI), Multiple sclerosis (MS) and neurodegenerative disorders such as Alzheimer’s disease or Parkinson’s disease [4–8].

Astrocytes, the most abundant and morphologically complex glial cells in CNS, play fundamental roles in brain homeostasis. These cells are crucial contributors to blood-brain barrier (BBB) formation and maintenance [9], while also serving as key components of the glymphatic system [10]. Under pathological conditions, including inflammation, neurodegenerative disease, and acute injury, astrocytes undergo “reactive” transformation, characterized by profound morphological, molecular, and functional alterations. These dynamic changes not only affect their homeostatic functions but also establish potential communication pathways with CNS-infiltrating immune cells [11].

The functional diversity of both neutrophils and astrocytes in CNS pathophysiology has become increasingly apparent. Neutrophils display context-dependent roles, ranging from neuroprotective to pro-inflammatory functions, while astrocytes exhibit remarkable plasticity in their inflammatory responses. The strategic positioning of astrocytes, maintaining direct contact with the cerebral vasculature, creates a unique interface for neutrophil-astrocyte communication [12]. This anatomical and functional relationship presents both opportunities and challenges for therapeutic intervention in CNS inflammatory conditions.

Here, we synthesize current understanding of neutrophil-astrocyte interactions in CNS inflammation, with particular emphasis on their bidirectional signaling mechanisms and therapeutic implications. We specifically address: (1) the distinct roles of neutrophils and astrocytes in CNS homeostasis and pathology; (2) the molecular and cellular interactions between neutrophils—including NETs—and reactive astrocytes across various disease models; and (3) the therapeutic potential of targeting these cellular interactions for treating neuroinflammatory disorders. By integrating recent advances in this field, we aim to provide a comprehensive framework for understanding how neutrophil-astrocyte crosstalk influences CNS inflammation and identify promising therapeutic strategies for neuroinflammatory diseases.

## Dual roles of neutrophils in CNS homeostasis

### Neutrophil origins and CNS barrier systems

Under physiological conditions, the CNS maintains a highly regulated immune environment, with neutrophils largely excluded from the brain parenchyma through a sophisticated barrier system. This system comprises the BBB, blood-meningeal barrier (BMB), and blood-cerebrospinal fluid (CSF) barrier, which collectively form a selective interface controlling immune cell trafficking [13, 14]. While the brain parenchyma proper remains largely devoid of neutrophils, specialized CNS compartments—including the CSF, meninges, and pia mater—harbor small populations of neutrophils and other immune cells that contribute to routine immune surveillance. Recent discoveries have identified substantial populations of neutrophils and other myeloid cells residing in the skull and vertebral bone marrow, establishing these structures as potential cellular reservoirs for rapid immune responses in the CNS [15]. This carefully maintained immune privilege can be dramatically altered under pathological conditions. During infection, trauma, ischemia, or hemorrhage, the tight regulation of neutrophil infiltration is disrupted, leading to substantial neutrophil accumulation within the brain parenchyma [16]. This shift from physiological exclusion to pathological infiltration represents a critical transition point in CNS inflammatory responses.

Recent advances have challenged traditional perspectives on neutrophil trafficking into the CNS by revealing dual origins of these cells: the classical blood-derived route and a newly discovered bone marrow source. While the hematogenous pathway of neutrophil infiltration has been well-documented [17], emerging evidence highlights an alternative route through direct ossified vascular channels that connect skull bone marrow to the CNS surface [18]. Single-cell RNA sequencing analyses have revealed that the skull and vertebrae house both developing and mature immune cells, mirroring the composition found in the tibial niche. Although these immune populations share similar transcriptional profiles across locations, skull-resident neutrophils exhibit distinct characteristics, including decreased expression of genes associated with myeloid cell differentiation [19]. This molecular signature suggests a specialized adaptation to the cranial microenvironment. Recent studies using fate-mapping and molecular tracing have further validated these findings [20].

This paradigm-shifting discovery demonstrates that the bone marrow niches within the skull and vertebrae, strategically positioned adjacent to the brain and spinal cord, serve as local reservoirs. These compartments can rapidly deploy monocytes and neutrophils to the meninges and CNS parenchyma during both homeostatic conditions and injury responses [15]. Notably, cranial neutrophils demonstrate unique functional properties, particularly in pathological conditions. For instance, following subarachnoid hemorrhage (SAH), skull marrow-derived neutrophils show an enhanced propensity for neutrophil extracellular traps (NETs) formation compared to their femoral counterparts. This increased NETosis potential persists in both healthy and disease states, suggesting an intrinsic functional specialization of cranial neutrophils [21]. Intriguingly, skull marrow-derived neutrophils appear to exhibit distinct functional properties, challenging the long-held assumption of blood-derived neutrophils as the primary mediators of CNS inflammation [18, 21, 22]. Similar functional distinctions have been observed in other pathological conditions [15, 20, 23]. The identification of this local neutrophil source raises fundamental questions about previous studies of neuroinflammation. Whether the documented effects of neutrophils in CNS pathologies primarily reflect the actions of skull marrow-derived rather than blood-derived populations remains to be determined. This uncertainty necessitates careful re-evaluation of existing literature and highlights the need for studies specifically distinguishing between these two neutrophil populations.

Neutrophils emerge as pivotal orchestrators in neuroimmune inflammation through diverse molecular mechanisms. Their protective functions are fundamentally anchored in sophisticated antimicrobial properties, predominantly mediated through specialized granules that serve as central effectors in both microbial elimination and inflammatory regulation [24]. A cornerstone of neutrophil antimicrobial activity lies in the nicotinamide adenine dinucleotide phosphate (NADPH) oxidase complex. This enzymatic assembly generates reactive oxygen species (ROS), which are instrumental in microbial killing. Notably, dysfunction or deficiency in this system frequently manifests as increased susceptibility to infections [25]. Complementing the NADPH oxidase system, chloride ion transport mechanisms represent another critical antimicrobial pathway. During phagocytosis, internalized chloride ions participate in the generation of hypochlorous acid (HOCl), a crucial halide in the myeloperoxidase (MPO)-mediated antimicrobial response. Perturbations in this pathway substantially compromise neutrophil bactericidal capacity [26]. The cystic fibrosis transmembrane conductance regulator (CFTR) serves as the primary conduit for chloride ion transport to neutrophil phagosomes, with CFTR-deficient neutrophils exhibiting marked impairment in their ability to eliminate Pseudomonas aeruginosa [27]. Beyond their direct antimicrobial functions, neutrophils demonstrate sophisticated immunomodulatory capabilities in adaptive immunity. They facilitate B cell responses through the secretion of key mediators including B cell activating factor (BAFF), a proliferation-inducing ligand (APRIL), and interleukin-21 (IL-21), which collectively enhance immunoglobulin class switching, somatic hypermutation, and antibody production [28]. Furthermore, neutrophils augment T cell responses in lymphoid tissues via upregulation of MHC-II, CD80, and CD86 [29]. They also participate in natural killer (NK) cell regulation through CD18/intercellular adhesion molecule-1 and CD18/intercellular adhesion molecule-3 (ICAM-3) dependent mechanisms [30, 31].

### Neutrophil functions in CNS homeostasis

While neutrophil recruitment and activation typically exert detrimental effects on neural tissue [2, 32], emerging evidence reveals an intriguing paradigm shift in our understanding of neutrophil-mediated neuroprotection. Particularly noteworthy are the zymosan-modulated Ly6G^low neutrophils, which demonstrate remarkable capacity for promoting both neuroprotection and regeneration across diverse neuronal populations, including retinal ganglion cells and dorsal root ganglia (DRG) neurons [33]. These specialized neutrophils execute their neuroprotective functions primarily through the secretion of key neurotrophic factors, notably Nerve Growth Factor (NGF) and Insulin-like Growth Factor 1 (IGF1). This finding holds particular promise for therapeutic interventions in spinal cord injuries, where DRG neurons frequently sustain damage. The potential exploitation of these neutrophil-specific properties could herald innovative therapeutic strategies aimed at enhancing nerve regeneration and functional recovery. Complementing these findings, N2 neutrophils have been identified as crucial mediators of neuroprotection through their secretion of immunomodulatory factors, specifically Transforming growth factor-beta (TGFβ) and IL-10 [34]. While the classification system for these neutrophil subtypes has been thoroughly documented and applied across numerous studies [35], recent groundbreaking research by Li et al. has unveiled distinct neuroprotective roles for neutrophils based on their anatomical origin [22]. This evolving understanding of immune cell functionality in neurological contexts continues to expand the therapeutic horizon for addressing previously intractable neurodegenerative conditions and neural injuries [33]. Such insights fundamentally reshape our approach to neuroimmunological interventions and highlight the sophisticated interplay between immune cells and neural tissue repair mechanisms.

The dichotomous nature of neutrophil function in the CNS presents a complex paradigm, where these cells exhibit both neuroprotective and neurotoxic effects. A fundamental aspect of neutrophil-mediated CNS pathology centers on their ability to compromise BBB integrity, which serves as a critical determinant of subsequent neurological outcomes. In the context of viral CNS infections, neutrophil-mediated BBB permeability modulation demonstrates this duality. Enhanced BBB permeability facilitates viral control by enabling antigen-specific lymphocyte infiltration to infection sites [36, 37], potentially augm enting viral clearance through adaptive immune responses [38]. Paradoxically, studies with neutrophil-depleted mice have shown reduced viral titers, highlighting the complexity of these interactions [39, 40]. The mechanism underlying BBB disruption involves neutrophil elastase, which degrades extracellular matrix proteins and cellular junction components, thereby increasing barrier permeability [41]. While increased BBB permeability can enhance immune surveillance and pathogen clearance during CNS infections, it simultaneously precipitates potentially devastating consequences. Cerebral edema, a life-threatening complication common to acute CNS disorders, represents one of the most significant manifestations of neutrophil-mediated BBB dysfunction [42, 43]. Recent breakthrough research has revealed that NETs degradation ameliorates hydrocephalus and brain injury by promoting lymphatic endothelial cell recovery and enhancing CSF drainage [44]. The relationship between neutrophils and cerebral edema remains controversial. While some studies demonstrate reduced edema formation and tissue damage following neutrophil depletion in murine traumatic brain injury models [45], others report no significant impact of neutropenia on hemispheric brain edema development [46]. Clinical evidence from Liu et al. correlates elevated neutrophil counts with malignant cerebellar edema (MCE) and poor outcomes in acute basilar artery occlusion (ABAO) patients [47]. Furthermore, the neutrophil-to-lymphocyte ratio has emerged as a predictive marker for perihematomal edema (PHE) following intracerebral hemorrhage [48, 49]. BBB dysfunction extends beyond edema formation, potentially triggering neuronal dysfunction, cognitive decline, and neurodegenerative processes [50]. Notably, neutrophils contribute directly to Alzheimer’s disease pathogenesis through lymphocyte function-associated antigen-1 (LFA-1) integrin interaction with ICAM-1 on BBB endothelial cells [7]. This molecular interface facilitates neutrophil CNS infiltration and subsequent neuroinflammation, potentially accelerating amyloid-beta (Aβ) plaque formation and disease progression. The impact of neutrophils on neural tissue extends to demyelination processes. Studies utilizing Cxcr2+/+ mice have demonstrated that CXCR2+ neutrophils significantly influence cuprizone-induced demyelination, with implications for axonal myelination and long-term recovery following traumatic brain injury [51]. This finding underscores the broad spectrum of neutrophil-mediated effects on CNS pathology and repair mechanisms.

The complex interplay between neutrophils and nociception represents a fascinating frontier in neuroimmunology. Pioneering work by Levine et al. [52] first demonstrated neutrophils’ hyperalgesic potential through elegant experiments utilizing N-formyl-methionyl-leucyl-phenylalanine and complement factor C5a injections into the dorsum of the paw. Notably, they observed that neutrophil depletion via hydroxyurea or methotrexate pretreatment effectively attenuated the hyperalgesic response. Neutrophil-mediated pain modulation operates through multiple pathways, including direct invasion of sensory ganglia - the primary structures responsible for nociceptive signal detection and transmission to the CNS. The relationship between neutrophils and pain perception presents an intriguing paradox: while substantial evidence supports their role in heightening pain sensitivity, with neutrophil depletion preventing chronic widespread pain development in murine models, other studies have documented either analgesic effects or no impact on nociception [53–55]. Current mechanistic insights reveal a complex molecular framework underlying neutrophil-mediated pain modulation. Key pro-nociceptive factors include Cathepsin E (CatE), ROS, and IL-1, which collectively contribute to mechanical allodynia development [56–58]. Conversely, neutrophil-derived products, specifically S100A8/S100A9 proteins and opioid peptides, demonstrate notable pain-resolving properties [59]. This dual capacity for both pain induction and resolution underscores the sophisticated nature of neutrophil-mediated nociceptive regulation.

### NETs formation and functions

Among the most intriguing capabilities of neutrophils is their ability to form NETs. These structures emerge through three distinct pathways: suicidal NETosis, vital NETosis, and mitochondrial NET formation (Mito-NETs) [60]. Suicidal NETosis represents a specialized form of cell death characterized by a precise sequence of cellular events. The process initiates with nuclear delobulation and nuclear envelope disassembly, progressing through loss of cellular polarization and chromatin decondensation. This pathway is typically triggered by phorbol myristate acetate (PMA) through activation of protein kinase C and the raf-MEK-ERK signaling cascade. NADPH oxidase plays a crucial role by facilitating neutrophil elastase translocation from cytosolic granules to the nucleus, where it mediates chromatin breakdown through histone cleavage. The culmination of this process occurs approximately 120 min post-initiation, when the neutrophil outer membrane ruptures to release the mature NET. Vital NETosis presents a distinct mechanism whereby NET formation occurs independently of cell death. This non-lytic process involves the coordinated expulsion of nuclear chromatin concurrent with granule protein release through degranulation. This pathway demonstrates particular sensitivity to microbial-specific molecular patterns recognized by host pattern recognition receptors. Notably, lipopolysaccharide (LPS) from gram-negative bacteria serves as a potent stimulus for rapid NET release. The process involves a sophisticated trafficking mechanism where DNA is transported from intranuclear vesicles to the extracellular space: DNA buds emerge from the nuclear membrane via vesicular structures, traverse the cytoplasm, and ultimately fuse with the plasma membrane, enabling NET release while maintaining cellular integrity [61–64]. The third pathway results in Mito-NETs, characterized by the release of extracellular traps composed of mitochondrial DNA decorated with cytoplasmic and granular proteins. While the molecular mechanisms underlying NET formation have been extensively documented in the literature [65], their diverse functional implications continue to emerge as an active area of investigation.

**Fig. 1:**
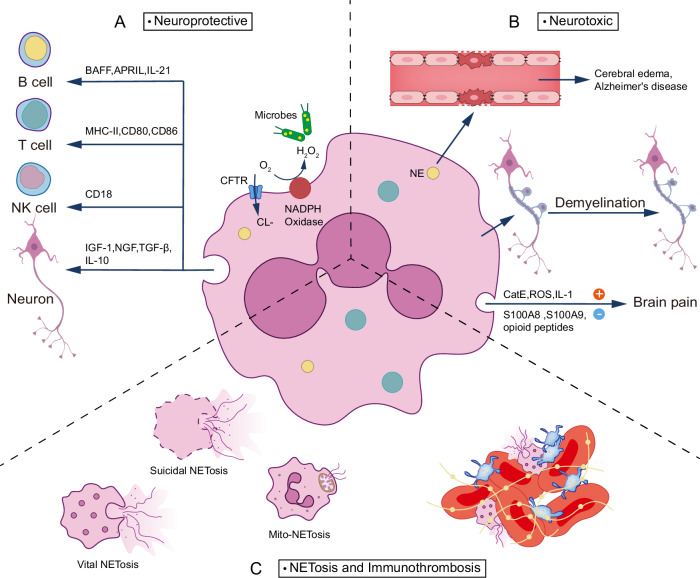
Physiological functions and signaling of neutrophils in CNS inflammation. Neutrophils in the CNS primarily play three roles. **A** Neutrophils play a protective role during neuroinflammation: antimicrobial, immune interactions and nerve regeneration. **B** Neutrophils play a neurotoxic role during neuroinflammation: damage to the BBB and the resulting cerebral edema, Alzheimer’s symptoms, demyelination, and pain allergy. **C** Neutrophils are involved in the construction of three types of NETosis as well as immunothrombosis.

NETs exhibit remarkable functional versatility, with their fundamental role being the establishment of physical barriers that constrain pathogen dissemination [66]. However, their involvement in pathological processes extends far beyond this primary function. In the context of thrombotic disorders, NETs serve as crucial mediators by initiating the coagulation cascade and providing an adhesive scaffold that facilitates platelet and erythrocyte attachment, thereby promoting thrombosis [67]. Their immunomodulatory capabilities are equally noteworthy, as excessive NET release can modulate inflammatory responses through the hydrolysis of cytokines and chemokines [68]. Of particular significance is the impact of NET-associated proteins on BBB integrity. The NET protein arsenal, including MPO, neutrophil elastase (NE), matrix metalloproteinases (MMP), and histones, can directly compromise BBB integrity and permeability, highlighting their potential role in neurological disorders. Beyond these primary functions, NETs have emerged as significant players in various pathological conditions. Their involvement spans autoimmune disorders [69, 70], metabolic diseases such as diabetes [71], and inflammatory conditions like periodontitis. Perhaps most intriguingly, mounting evidence suggests their participation in cancer progression and metastatic spread, underscoring the far-reaching implications of NET biology in human disease (Fig. 1).

## Reactive astrocytes: dual roles in CNS function

### Astrocyte phenotypes and reactivity

Astrocytes, often referred to as the “stars of the brain,” represent a specialized glial cell population that numerically dominates neurons by a factor of five and permeates the entire brain parenchyma. These cells serve as master regulators of brain homeostasis and neuronal function. In response to CNS injury or disease, astrocytes undergo a remarkable transformation termed “reactive astrocytosis”—a heterogeneous process encompassing molecular, cellular, and functional modifications that may culminate in glial scar formation. The advent of GFAP antibodies marked a significant milestone in identifying astrocyte reactivity, predicated on the correlation between elevated GFAP expression and the transition from quiescent to reactive states. While this paradigm proves valid in numerous scenarios, it’s important to note that GFAP expression exhibits regional heterogeneity in the healthy CNS and varies according to the nature and location of the triggering stimulus. Recent advances in genomic analysis have revolutionized our understanding of astrocyte reactivity, particularly through the identification of distinct reactive phenotypes. The classical dichotomy comprises the neurotoxic or pro-inflammatory (A1) and neuroprotective or anti-inflammatory (A2) states [72]. However, this binary classification represents an oversimplification of a more complex reality. For instance, normal aging processes, characterized by pro-inflammatory microglia and elevated C1q protein levels, can trigger A1 astrocyte formation. Similar A1 responses are observed in various neurodegenerative conditions, including Alzheimer’s disease (AD) [73], Huntington’s disease (HD), Parkinson’s disease (PD), amyotrophic lateral sclerosis (ALS), and MS, all of which demonstrate positive immunoreactivity for C3 and other A1-specific markers. A2 astrocytes play particularly crucial roles in cerebral ischemia and infarction [74], and their presence has been documented in Alzheimer’s disease [75]. Beyond these canonical states, novel astrocyte phenotypes continue to emerge. Notable examples include the C3+ PrPSc+ astrocyte subtype identified in prion disease models and the distinctive AxD astrocyte subtype associated with Alexander disease (AxD). Intriguingly, while these subtypes share some transcriptional similarities with A1-like astrocytes, they maintain distinct molecular signatures [76, 77]. The complexity of astrocyte heterogeneity is further exemplified in spinal cord injury (SCI), where researchers have identified six transcriptionally distinct astrocyte subtypes [78]. While we currently employ these classical polarization states as a framework for understanding astrocyte reactivity in common pathological models, the future lies in single-cell genomic analysis coupled with multidimensional data integration and co-clustering approaches. These advanced methodologies promise to unravel the full spectrum of astrocyte heterogeneity and their functional implications in health and disease.

Reactive astrocytes exhibit remarkable functional diversity in neuroinflammation, often displaying contradictory roles across different studies. Their responses can vary significantly depending on the severity of CNS damage [79]. Among their various manifestations, astrocytic scarring stands as perhaps the most well-characterized feature of reactive astrogliosis. The impact of astrocyte scarring on CNS axon regeneration has emerged as a subject of fascinating debate, with two competing theories currently dominating the field. In a groundbreaking study, Anderson et al. demonstrated that scar-forming astrocytes actually facilitate and support robust CNS axon regeneration. This supportive function operates through the upregulation of specific growth-promoting molecules, notably particular chondroitin sulfate proteoglycans (CSPGs, specifically CSPG4 and CSPG5) and laminins. Their work revealed that axonal regeneration across CNS lesions becomes possible when two conditions are met: the presence of scar-forming astrocyte bridges and genetic activation of axonal outgrowth programs in mature neurons. This occurs despite CSPG production being traditionally viewed as the primary mechanism inhibiting axon regeneration [80]. The contrasting theory posits that glial scarring constitutes a formidable barrier to nerve regeneration. According to this model, reactive astrocytes secrete an array of growth inhibitors that actively suppress axon regeneration through the Rho/ROCK signaling pathway. These inhibitory factors include CSPGs, Class 3 semaphorin proteins (SEMA3) and their high-affinity receptor neuropilin 1, ephrin-B2 and its receptor EPHB2, as well as Slit proteins in conjunction with glypican 1 receptors [81].

### Functional diversity and synaptic regulation

The complexity of reactive astrocytes extends to their critical role in synaptic regulation. A key mechanism involves thrombospondins (TSP), particularly TSP1 and TSP2, which are upregulated in reactive astrocytes and play instrumental roles in synaptic plasticity and repair processes. These astrocyte-derived factors orchestrate crucial aspects of synaptic remodeling in the injured brain. Intriguingly, reactive astrocytes participate in both synapse formation and elimination—a delicate balance that shapes neural circuit refinement after injury. The elimination process relies heavily on phagocytic receptors, notably Mer tyrosine kinase (MERTK) and MEGF10, which facilitate the removal of redundant or damaged synaptic connections. However, these receptors typically show reduced expression in reactive astrocytes, potentially compromising synaptic pruning efficiency. This disruption can lead to suboptimal neural network reorganization and impaired recovery processes [82–84]. The relationship between reactive astrocytes and synapse formation presents a double-edged sword. While enhanced synaptogenesis might seem beneficial, excessive synapse formation can trigger pathological consequences. Such overabundance of synaptic connections can disrupt normal neuronal signaling cascades, potentially manifesting as seizures or neuropathic pain [85, 86]. Further complexity emerges at the transcriptomic level, where reactive astrocytes exhibit dynamic regulation of synaptic molecules, including SPARCL1 [87], GYP4 and GYP6 [88]. Paradoxically, these changes often result in diminished synaptogenic support, compromising the brain’s ability to form compensatory synaptic connections following injury. This reduced capacity for synaptic support may impede neural network reconstruction, potentially leading to more severe functional deficits in both cognitive and motor domains.

Additionally, Reactive astrocytes exhibit a remarkable duality in their interactions with neurons, orchestrating both neuroprotective and neurotoxic effects depending on the contextual environment. Seminal work by Bush et al. demonstrated the essential neuroprotective role of reactive astrocytes, revealing rapid neuronal death following astrocytic ablation in mice [89]. Further supporting this protective function, Aguirre-Rueda D documented how astrocytes shield neurons from Aβ1-42 peptide-induced neurotoxicity through modulation of key metabolic regulators, specifically by enhancing TFAM and PGC-1 while suppressing PPAR-γ and SIRT-1 [90]. Intriguingly, these reactive glial cells possess the capacity for direct reprogramming into functional neurons in vivo, offering a potentially transformative approach for treating brain injury and disease [91]. The neurotoxic aspects of reactive astrocytes manifest through three distinct mechanisms. First, specific reactive astrocyte subsets secrete soluble toxins that selectively target CNS neurons and mature oligodendrocytes while sparing other cell types [74]. This toxicity extends to the overproduction of conventional neuromodulators and nutrients, notably NO [11, 92] and saturated lipids [93]. Second, disruption of glutamate homeostasis emerges as a critical pathway to neuronal injury. Astrocytes typically maintain glutamate balance through high-affinity transporters EAAT1 (encoded by SLC1A3) and EAAT2 (encoded by SLC1A2) [94], which are fundamental for preventing excitotoxicity. However, reactive astrocytes can compromise this protective mechanism through enhanced glutamate release and altered reuptake dynamics, potentially triggering excitotoxic neuronal death [95]. The third mechanism involves the deterioration of astrocytic nutritional support [96]. Under physiological conditions, astrocytes maintain neuronal excitability through sophisticated K+ homeostasis, facilitated by diverse K+ channels [3]. Dysfunction of specific channels, particularly Kir4. 1 (inwardly rectifying K+ channel), can elevate extracellular K+ and enhance SPN spiking, potentially precipitating excitotoxicity [97, 98]. Similarly, Kir6. 1 impairment can exacerbate dopaminergic neuronal damage. While evidence supports all three mechanisms, their precise relationship to distinct reactive astrocyte subsets remains an active area of investigation, highlighting the complex heterogeneity of these cells [99].

Within CNS innate immunity, astrocyte reactivity plays a fundamental role. These reactive astrocytes engage in sophisticated interactions with glial and peripheral immune cells [11], orchestrating inflammatory responses following various CNS insults, including trauma and stroke. Paradoxically, they can also restrict inflammatory spread through anti-inflammatory signaling cascades and barrier formation [100]. This immunomodulatory versatility extends to tumor microenvironments, where reactive astrocytes can inappropriately suppress local inflammation via Stat3-mediated mechanisms [101].

The BBB represents a sophisticated defense system crucial for CNS homeostasis, excluding potentially harmful plasma components, blood cells, and pathogens. Astrocytes serve as key mediators of neurovascular communication, with their specialized endfeet forming an integral component of the neurovascular unit (NVU), alongside brain endothelial cells, vascular smooth muscle cells (VSMCs), pericytes, and vascular basement membranes [102]. Under pathological conditions, astrocytic phenotypic alterations significantly impact BBB integrity. For instance, in canine demyelinating disease, reactive astrocytes exhibit progressive loss of AQP-4 expression [103]. Given that AQP-4 is predominantly expressed in perivascular glial processes and ependymal cell linings, its depletion serves as a critical indicator of barrier dysfunction, potentially compromising CNS homeostasis. Furthermore, reactive astrocytes directly modulate BBB integrity through diverse mechanisms. In SAH models, they can compromise BBB integrity through SPARC-mediated activation of integrin αVβ3 signaling in endothelial cells [104]. Conversely, these cells also demonstrate protective functions through increased osteopontin (OPN) expression [105], which promotes BBB recovery following SAH.

**Fig. 2:**
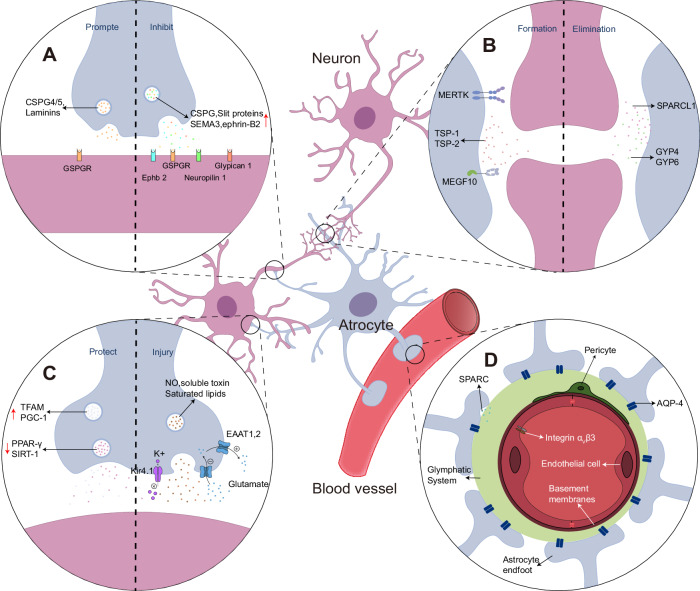
Physiological functions and signaling of reactive astrocytes in CNS inflammation. Astrocytes in the CNS play four main roles: **A** Glial scar formation by astrocytes play a dual role in axons during neuroinflammation. **B** Reactive astrocytes play a dual role in synaptic formation during neuroinflammation. **C** Reactive astrocytes play a dual role in neurons itself during neuroinflammation. **D** Reactive astrocytes affect BBB and glymphatic system function.

The glymphatic system, characterized by periarteriovenous spaces formed by astrocytic endfeet, exhibits remarkably dense AQP-4 distribution [10]. Consequently, pathology-induced changes in AQP-4 polarization within reactive astrocytes can profoundly influence glymphatic function. Recent investigations by Feng et al. have revealed differential AQP-4 polarization patterns between astrocyte phenotypes, with A2 astrocytes showing enhanced polarization compared to A1 variants. Notably, AQP-4 depolarization significantly compromises glymphatic system efficiency in clearing Aβ and tau proteins [106], highlighting the critical role of astrocytic polarization in maintaining brain homeostasis (Fig. 2).

## Astrocyte reactivity orchestrates neutrophil-mediated innate immunity

### Neutrophil-astrocyte interactions and signaling mechanisms

The intricate interplay between neutrophils and reactive astrocytes within the CNS represents a complex paradigm of cellular communication, characterized by both synergistic and antagonistic functions. This functional duality creates a sophisticated regulatory network when these cells coexist within the same microenvironment. Understanding the molecular dialogue between neutrophils and reactive astrocytes has emerged as crucial for deciphering both the evolution and resolution of CNS inflammation, while simultaneously offering promising therapeutic targets for neurological and neuroimmunological disorders. The bidirectional communication between reactive astrocytes and neutrophils orchestrates CNS inflammatory responses through an elaborate network of cytokines and inflammatory mediators. While substantial evidence demonstrates that reactive astrocytes can modulate neutrophil function and activity through diverse mechanisms, several critical aspects remain to be fully elucidated. These include the precise molecular mechanisms underlying their interactions, the regulatory pathways governing their communication, and how these dynamics adapt across different pathological contexts. Such knowledge gaps represent both challenges and opportunities for future therapeutic interventions in CNS disorders.

Reactive astrocytes emerge as critical modulators of neutrophil activity during neuroinflammation through their diverse secretory repertoire of cytokines and inflammatory mediators. Pioneering in vitro studies by Xie et al. revealed a nuanced regulatory system wherein astrocytes orchestrate neutrophil functions through both direct and indirect cellular interactions [107]. Direct cell-cell contact results in a complex modulation of neutrophil behavior: suppressing apoptosis, respiratory burst, and degranulation, while simultaneously enhancing phagocytic capacity and pro-inflammatory cytokine production. These interactions manifest in striking morphological transformations of neutrophils, from their characteristic round, flattened resting state to a distinctive loosely adherent configuration. Through paracrine signaling, astrocytes demonstrate equally sophisticated control over neutrophil function, modulating cell death pathways, augmenting phagocytosis and respiratory burst, while attenuating degranulation. Notably, astrocytes amplify key signaling cascades in neutrophils, including Akt, Erk1/2, and p38 pathways [108]. While these in vitro findings provide crucial mechanistic insights, the complexity of in vivo astrocyte-neutrophil interactions, embedded within the intricate cellular microenvironment of the CNS, demands further investigation. Recent advances have begun to illuminate these complex dynamics. Lai et al. demonstrated that endothelial-reactive astrocyte signaling in Cerebral Cavernous Malformation orchestrates immune cell recruitment, with notable accumulation of neutrophils along vessel walls and within thrombi [109]. This mechanistic understanding gained substantial support from Ji et al.’s observations [110], who revealed a critical relationship between astrocyte density and BBB integrity. Specifically, in regions of low astrocytic density, LPS activation triggers a more severe disruption of the BBB in the substantia nigra pars compacta (SNpc) compared to the cortex. This enhanced BBB compromise facilitates robust neutrophil infiltration into the SNpc, potentially initiating a detrimental feedback loop. The resulting self-perpetuating cycle encompasses increased BBB permeability, sustained neutrophil infiltration, and progressive damage to both astrocytes and endothelial cells, ultimately amplifying further neutrophil recruitment and tissue damage. These findings highlight the intricate interplay between astrocyte density, BBB integrity, and neutrophil infiltration, prompting researchers to delve deeper into the underlying molecular mechanisms. Of particular interest is how astrocytes, through their diverse molecular repertoire, orchestrate neutrophil recruitment under different pathological conditions. This line of investigation has proven especially fruitful, as the molecular mechanisms underlying astrocyte-mediated neutrophil recruitment have been progressively unveiled across various disease models. In West Nile virus infection, Kuwar et al. identified astrocyte-derived S100B as a key mediator of Mac-1-dependent neutrophil migration to compromised BBB sites, potentially operating through RAGE-mediated upregulation of adhesion molecules [110, 111]. This chemotactic paradigm extends to other viral encephalitides, as evidenced in HSV-1 infection, where astrocytic CXCL1 signaling through CXCR2 governs neutrophil trafficking and BBB integrity [37]. Complementary findings from Isabelle Pineau’s group established the critical role of MyD88/IL-1R1 signaling in regulating astrocytic chemokine expression (KC/CXCL1 and MIP-2/CXCL2) and subsequent neutrophil recruitment in spinal cord injury [112]. The scope of astrocyte-neutrophil interactions extends beyond viral pathologies. In HIV latency studies, Proust et al. demonstrated that bryostatin-1-treated astrocytes promote both neutrophil transmigration and NETosis through IL-8 secretion [113]. Even in chronic neurodegenerative conditions like ME7 prion disease, astrocytes display enhanced CXCL1 responses to IL-1β stimulation, facilitating neutrophil recruitment [114]. Recent investigations in acute brain injury models have revealed additional molecular mechanisms. Jing Huang et al. identified a KDM4A/NF-κB-dependent pathway regulating astrocytic CXCL1 expression in ischemic injury [115]. Furthermore, astrocyte-derived extracellular vesicular C-143-3p targets ATP6V1A in brain microvascular endothelial cells [116], modulating endothelial adhesion molecule expression and neutrophil transmigration [117]. In traumatic brain injury, sICAM-1 orchestrates neutrophil chemotaxis through src tyrosine kinase and p42/44 MAPK activation, culminating in astrocytic MIP-2 production [118].

The temporal dynamics of astrocyte-mediated neutrophil recruitment demonstrate intriguing complexity, governed by circadian rhythms. The core clock gene Bmal1 in astrocytes exhibits consistent expression patterns across CNS regions, peaking around zeitgeber time 12 (ZT12, 12 h after lights on) [119]. This temporal regulation aligns with the diurnal variations in circulating neutrophil populations, where aged neutrophils peak at approximately ZT5, while “fresh” neutrophils predominate at ZT13 [120]. Astrocytic secretion of inflammatory mediators, including IL-6, IL-1β, and CCL2, demonstrates marked dark-phase elevation, contributing to enhanced blood-brain barrier permeability [121] and subsequent neutrophil recruitment [122–124]. Moreover, astrocytic Bmal1 emerges as a critical regulator of BBB homeostasis through its influence on endfoot AQP-4 polarization, with implications for BBB permeability and glymphatic system function, potentially modulating neutrophil trafficking [125, 126]. Although direct studies of lymphatic function in circadian rhythm disruption models remain limited, shift workers consistently exhibit elevated markers of systemic inflammation [126]. Under physiological conditions, these diurnal fluctuations in astrocyte-derived inflammatory signals and BBB permeability remain modest, with minimal impact on neutrophil trafficking. However, pathological conditions—including neuroinflammation, neurodegeneration, and CNS injuries—can dramatically amplify these circadian effects, leading to clinically significant alterations in BBB integrity and neutrophil recruitment [114, 125, 127, 128]. This is exemplified by Chi3l1/YKL-40, a molecule under circadian clock control, whose genetic deletion (BRP-39−/−) results in exacerbated and sustained immune infiltration [129, 130]. Similarly, Bmal1-deficient astrocytes display heightened inflammatory responses [125], underscoring the importance of intact circadian regulation in maintaining appropriate neuroimmune responses.

### AQP-4 regulation and future directions

In contrast to the pro-infiltration mechanisms, compelling evidence suggests that reactive astrocytes can also act as gatekeepers of the CNS parenchyma. A seminal study by Voskuhl et al. in 2009 demonstrated that reactive astrocytes form scar-like perivascular barriers, effectively restricting leukocytes (including neutrophils) to perivascular spaces and limiting their parenchymal infiltration during experimental autoimmune encephalitis (EAE) [131]. Further complexity in astrocyte-mediated neutrophil regulation has emerged from recent studies of Status epilepticus (SE). Kim et al. unveiled a novel mechanism operating independently of vascular permeability, wherein the non-integrin 67-kDa laminin receptor (67LR)-ERK1/2 (extracellular signal-regulated kinase 1/2)-MIP-2 (macrophage inflammatory protein-2) signaling axis in astrocytes serves to suppress neutrophil infiltration. Mechanistically, 67LR neutralization enhanced ERK1/2 phosphorylation in astrocytes, while co-treatment with U0126, an ERK1/2 inhibitor, effectively attenuated the 67LR neutralization-induced increase in phospho-ERK1/2 levels [132]. These findings underscore the remarkable complexity of reactive astrocyte-mediated regulation of CNS leukocyte trafficking. Rather than following a simple binary pattern, astrocytes appear to orchestrate context-dependent responses, either promoting or inhibiting neutrophil migration depending on the specific pathological scenario. This functional plasticity highlights the need for careful consideration of disease-specific contexts when developing therapeutic strategies targeting astrocyte-mediated immune responses.

**Fig. 3:**
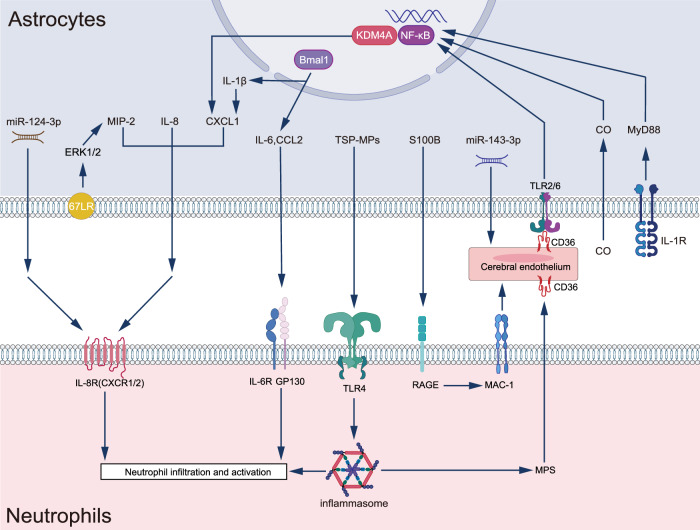
Reactive astrocytes regulate neutrophils in vivo. Astrocytes regulate neutrophils. In vivo, astrocytes can influence neutrophil infiltration and activation through a variety of pathways.

Beyond their role in neutrophil trafficking, reactive astrocytes emerge as essential modulators of neutrophil phenotype and function. Li et al. revealed an sophisticated mechanism whereby astrocyte-derived extracellular vesicles carrying miR-124-3p suppress MPO+ neutrophil expression in neonatal hypoxic-ischemic brain injury, conferring neuroprotection [133]. A particularly striking example of astrocyte-neutrophil communication comes from studies of carbon monoxide (CO) poisoning. Ruhela et al. [134] uncovered a complex feed-forward inflammatory cascade initiated by astrocytic NF-κB activation. This pathway triggers the production of thrombospondin-1 (TSP-1)-containing microparticles (MPs), which enter systemic circulation via cervical lymphatics. These MPs engage multiple receptors on neutrophils—including Toll-like receptor-4, CD47, and integrins—promoting NLRP3 inflammasome assembly. This activation leads to the generation of Ly6G-expressing blood-derived MPs, amplifying oxidative stress. Furthermore, circulating MPs can interact with endothelial CD36, sustaining astrocytic NF-κB activation and AQP-4 dysregulation, thus perpetuating the inflammatory cycle [135]. While neutrophil activation is documented in acute CO poisoning patients [136], the precise conservation of these mechanistic details in human pathology awaits confirmation. These findings illuminate the sophisticated regulatory networks through which reactive astrocytes modulate neutrophil populations, underscoring their central importance in neurological disease pathogenesis. Future investigations should leverage advanced spatial and temporal approaches to decode these intricate interactions, unravel the mechanisms underlying their context-dependent plasticity, and exploit this knowledge for therapeutic benefit. Understanding how environmental cues drive astrocytes to either exacerbate or resolve neuroinflammation may reveal novel therapeutic targets for neurological disorders (Fig. 3).

## Neutrophil-derived factors orchestrate astrocyte heterogeneity and function

### Neutrophil-astrocyte interactions: barrier crossing and molecular mechanisms

Reactive astrocytes and neutrophils engage in a bidirectional regulatory relationship. Under physiological conditions, the CNS maintains immune privilege through specialized barrier structures, including the blood-brain barrier and blood-cerebrospinal fluid barrier. However, during CNS perturbations, neutrophils can traverse both intact and compromised barriers, subsequently orchestrating astrocyte fate under various pathological conditions. While the traditional barrier-crossing routes are well-documented, an alternative pathway through skull bone marrow-meningeal connections (SMCs) remains largely unexplored. Regardless of the entry mechanism [18, 137], these trafficking pathways are fundamental for enabling neutrophil-astrocyte interactions. During neuroinflammation, astrocytes constitute the primary CNS-resident cells encountered by infiltrating neutrophils. Recent mechanistic investigations have begun to decipher the molecular dialogue between these cell types. Notably, neutrophils drive astroglial differentiation of human neural stem cells through the synthesis of complement components C1q and C3a [138]. Furthermore, neutrophil-derived TNF-α promotes astrocyte differentiation through STAT3 signaling activation, leading to expanded astrocytic and neuronal populations [139, 140]. These molecular mechanisms underscore the profound capacity of neutrophils to modulate astrocyte biology.

Neutrophil regulation of astrocytes encompasses multiple mechanistic pathways, with mounting evidence supporting direct modulation of astrocyte reactivity. In vitro investigations by Moreno-Flores et al. revealed that neutrophil-astrocyte co-culture induces profound alterations in astrocytes, including morphological restructuring, stromal detachment, and cellular death [141]. The functional significance of these interactions has been corroborated in vivo, where Stirling et al. demonstrated that neutrophil depletion via anti-Ly6G/Gr-1 antibodies (reducing neutrophil rolling and adhesion by >90%) attenuated astrocyte reactivity following SCI, ultimately compromising behavioral and histological recovery [142]. While the precise mechanisms remained undefined, this study established the beneficial role of neutrophil-astrocyte interactions in SCI recovery. Recent advances have uncovered additional molecular mediators of this cellular crosstalk. Notably, Dectin-1 signaling from CNS-infiltrating myeloid cells, including neutrophils, facilitates myeloid cell-astrocyte communication through the Osm-OsmR axis in EAE. This interaction, coupled with astrocytic gp130-OsmR heterodimer formation, confers protection in EAE [143, 144]. Mülling et al. further characterized neutrophil-dependent modulation of astrocytes through two distinct parameters: while neutrophil depletion did not affect overall astrocyte numbers, it significantly reduced glial activation and protein expression levels, as evidenced by diminished Iba-1 and GFAP staining intensity [145]. Mechanistically, infiltrating neutrophils influence astrocyte phenotype through MPO-mediated signaling, triggering early reduction in GFAP expression, astrocyte loss, and enhanced Vimentin-GFAP colocalization [146]. Importantly, while neutrophil depletion does not alter total astrocyte numbers, reactive astrocytosis exhibits spatial correlation with regions of robust neutrophil infiltration [147].

### AQP-4 regulation and future perspectives in neuroinflammation

Neutrophils modulate the expression and function of astrocytic AQP-4. AQP-4, predominantly localized to astrocytic endfeet, serves as a critical regulator of central nervous system water homeostasis and is intimately linked to cerebral edema pathophysiology. Seminal work by Haj-Yasein et al. demonstrated that glial-specific AQP-4 deletion attenuates blood-brain water uptake [148]. Complementary studies in AQP-4-deficient animals revealed paradoxically elevated brain water content and impaired parenchymal extracellular fluid clearance at the blood-brain barrier compared to wild-type counterparts [149]. In the context of neuromyelitis optica (NMO), Saadoun et al. uncovered a crucial relationship between neutrophil activity and AQP-4 integrity. Their investigations revealed that neutropenia models exhibited reduced AQP-4 loss, persisting for at least 12 h, while neutrophilia exacerbated AQP-4 damage. Through elegant mechanistic studies employing NE, its inhibitor Sivelestat, and cathepsin G modulators, they established NE as the principal mediator of this pathway, underscoring the fundamental role of neutrophil-derived proteases in NMO pathogenesis [150]. Recent investigations have further elucidated that neutrophils synergize with anti-AQP-4 antibodies to promote astrocyte destabilization and glutamate transporter dysfunction in neuromyelitis optica spectrum disorder (NMOSD) [151].

**Fig. 4:**
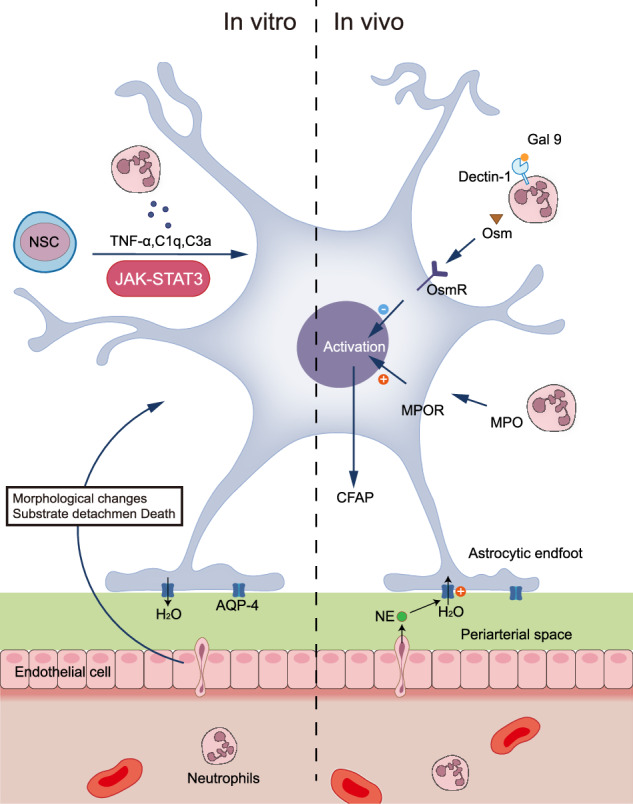
Neutrophils modulate astrocytes heterogeneity and functions. Neutrophils regulate astrocytes. In vitro, neutrophils can affect the survival of astrocyte formation. In vivo, neutrophils can influence astrocytes activation through a variety of pathways.

Astrocyte polarization represents a critical regulatory mechanism in neuroinflammation, manifesting as either neurotoxic pro-inflammatory (A1) or neuroprotective anti-inflammatory (A2) phenotypes. While extensive literature has documented these distinct activation states, compelling evidence indicates that A1 astrocytes are predominantly induced through microglial activation [74]. However, the potential role of neutrophils in modulating astrocyte phenotype remains largely unexplored. Critical questions persist regarding whether neutrophils can directly influence astrocyte phenotypic transitions, analogous to microglial-mediated effects, or whether they operate through synergistic interactions with microglia to orchestrate astrocyte polarization. Future investigations will require sophisticated methodological approaches to decipher these complex cellular interactions. Integration of cutting-edge technologies, including in vivo imaging and organoid co-culture systems, combined with emerging interdisciplinary approaches such as immunometabolomics, will be essential to elucidate the intricate mechanisms governing neutrophil-astrocyte communication. Such comprehensive understanding could ultimately inform precision therapeutic strategies for neuroinflammatory disorders, potentially through targeted modulation of the neutrophil-A1/A2 axis (Fig. 4).

### Neutrophil extracellular traps and reactive astrocytes crosstalk

NETs represent a sophisticated innate immune defense mechanism, characterized by the strategic deployment of web-like chromatin structures in response to diverse pathological stimuli. These intricate fibrous networks, generated through regulated neutrophil activation, comprise extracellular cell-free DNA scaffolds decorated with an arsenal of bioactive components. The molecular constituents include histones, NE, MPO, serine proteases, calcineurin, antimicrobial peptides, defensins, and actin, along with numerous yet-to-be-characterized bioactive molecules. Beyond their established role in neuroinflammation, emerging evidence suggests that NETs serve as crucial mediators of astrocyte function and regulation during neuroinflammatory processes.

**Fig. 5:**
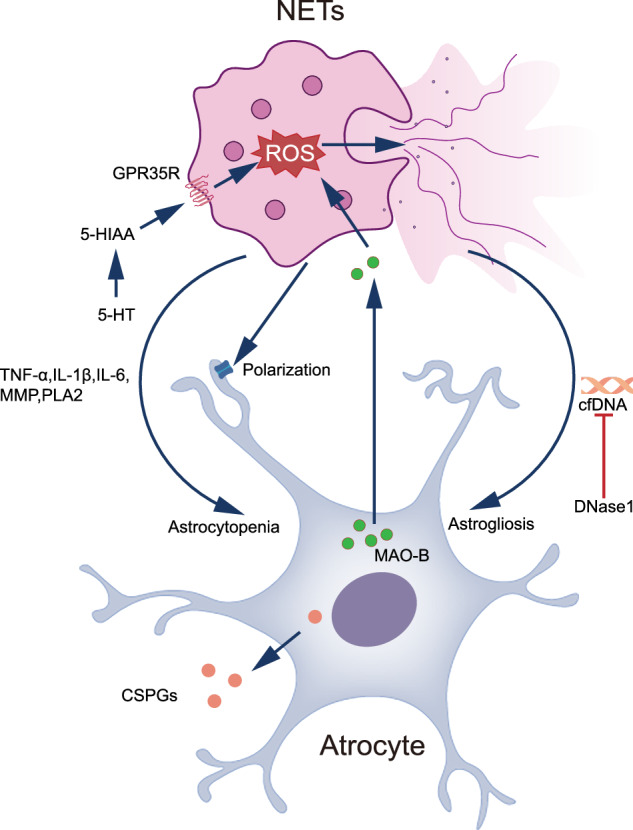
NETs and astrocyte interplay. Bidirectional regulation of nets and astrocytes. Astrocytes mainly affect NETS production by activating neutrophils to produce ROS. Nets can affect astrocyte numbers and AQP-4 on the endfoot.

Recent advances in neuroimmunology have uncovered a sophisticated regulatory network between NETs and astrocyte function in the central nervous system. Recent studies have demonstrated that NETs contribute to astrocytopenia through a cascade of events involving elevated pro-inflammatory cytokines (TNF-α, IL-1β, and IL-6) in the brain, subsequently triggering microglial activation. Kong et al. provided compelling evidence that PAD4 ablation, which reduces histone H3 citrullination (CitH3) and prevents chromatin decondensation-mediated NETs formation [152], significantly attenuates astrocyte loss [150]. Intriguingly, Xie et al. unveiled the crucial role of cell-free DNA (cfDNA), the structural backbone of NETs, in astrocyte dynamics. Their findings revealed that DNase1-mediated cfDNA degradation reduces periventricular astrocyte proliferation, suggesting that NET formation promotes astrocyte proliferation in this region [153]. This apparent paradox in NET-mediated astrocyte regulation warrants further mechanistic investigation to optimize therapeutic strategies. Additional studies have shown that NET dissolution by DNase I influences perivascular AQP-4 polarization, albeit with modest effects [154]. Moreover, NETs facilitate reactive astrocyte formation and promote CSPG production in spinal cord injury, contributing to glial scar formation [155]. Therapeutic approaches targeting NET dynamics, including PAD4 inhibition or DNase1-mediated NETs degradation, show promise in treating central nervous system disorders characterized by aberrant reactive astrocyte responses. The potential therapeutic value of NADPH oxidase inhibitors, which suppress neutrophil ROS production [156], merits further investigation in this context. Conversely, astrocytes exhibit regulatory effects on NET formation. Li et al. demonstrated that administration of the MAO-B inhibitor selegiline, alone or in combination with CI-amidine, suppresses NET formation, thereby attenuating neuroinflammation and inflammatory pain. This effect is attributed to the dual action of MAO-B inhibitors in suppressing both intracellular 5-HT metabolism to 5-HIAA and ROS production in reactive astrocytes [157]. These findings underscore the potential therapeutic applications of NADPH oxidase inhibitors in central nervous system disorders, particularly in Parkinson’s disease [158, 159]. While these studies establish the interconnected nature of NETs and astrocytes, the molecular mechanisms underlying their interactions require further elucidation. This emerging field presents promising therapeutic opportunities while highlighting the need for more comprehensive mechanistic investigations (Fig. 5).

### Therapeutic implications and future directions of neutrophil-astrocyte interactions

The intricate interplay between neutrophils and astrocytes represents a compelling therapeutic target across diverse neurological disorders. Recent advances in understanding these cellular interactions have unveiled multiple intervention points, each offering distinct therapeutic possibilities. At the forefront, strategies targeting neutrophil trafficking have shown considerable promise: CXCR2 antagonism effectively attenuates CNS-directed neutrophil migration during neuroinflammation [160] and ischemic stroke [161], while modulation of the 67LR-ERK1/2-MIP-2 axis through ERK1/2 inhibitors such as U0126 has demonstrated efficacy in reducing neutrophil infiltration in epileptic conditions [132]. Astrocyte-directed interventions have emerged as another crucial therapeutic avenue. Notably, suppression of the NF-κB pathway diminishes TSP-1 microparticle generation, while targeted delivery of miR-124-3p shows promise in attenuating MPO-positive neutrophil expression following ischemic insult [133]. The therapeutic landscape has been further enriched by approaches targeting neutrophil extracellular traps (NETs), encompassing DNase-mediated NET degradation, neutrophil elastase inhibition via Sivelestat in neuromyelitis optica, and innovative combination therapies coupling MAO-B inhibition (selegiline) with NET suppression (CI-amidine) [157]. Intriguingly, chronotherapeutic considerations have gained traction, with mounting evidence supporting the optimization of intervention timing based on circadian fluctuations in astrocyte and neutrophil activity. The manipulation of molecular timekeepers, particularly Bmal1, presents opportunities for modulating both inflammatory responses and blood-brain barrier dynamics. This temporal dimension adds complexity to the already multifaceted challenge of developing disease-specific and phase-appropriate therapeutic strategies. Despite these promising advances, the path to clinical implementation remains fraught with challenges. Critical hurdles include the development of robust biomarkers, precise therapeutic timing, comprehensive safety profiling, and the engineering of targeted delivery systems. Looking ahead, the field must prioritize the development of cell-specific targeting approaches, innovative drug delivery platforms, and predictive biomarkers, while simultaneously advancing rigorous preclinical and clinical validation studies. As our understanding of neutrophil-astrocyte dynamics continues to evolve, these cellular interactions increasingly appear central to CNS inflammatory conditions. The convergence of mechanistic insights with therapeutic innovation suggests we are entering a promising era in the treatment of neurological disorders, though careful validation and optimization of these approaches remains paramount.

## Concluding remarks

The intricate crosstalk between neutrophils and reactive astrocytes emerges as a pivotal regulatory mechanism in CNS inflammation, orchestrated through complex signaling networks between resident and recruited cells. Despite recent advances, critical knowledge gaps persist. Several fundamental questions warrant further investigation: (1) the molecular mechanisms governing neutrophil-mediated astrocyte phenotype modulation; (2) the specific neutrophil-derived factors that regulate astrocytic AQP-4 polarization; and (3) the NET components responsible for modulating astrocyte AQP-4 polarity. Elucidating these neutrophil-astrocyte interactions holds promise for therapeutic intervention. Such insights are advancing our understanding of neural cell networks at a systems level [162], ultimately paving the way for novel therapeutic strategies in treating CNS inflammatory disorders. ultimately paving the way for novel therapeutic strategies in treating CNS inflammatory disorders.
